# Gut Dysbiosis-Mediated Major Depressive Disorder: A Review of Pathogenic Mechanisms and Potential Therapeutic Strategies

**DOI:** 10.3390/cells15110972

**Published:** 2026-05-25

**Authors:** Muhammad Sohail Khan, Muhammad Faizan, Gabsik Yang, Ki Sung Kang

**Affiliations:** 1 College of Korean Medicine, Gachon University, 1342 Seongnam-daero, Seongnam 13120, Republic of Korea; sohail@gachon.ac.kr; 2 Department of Animal Reproduction, Faculty of Veterinary Sciences, The University of Agriculture, Peshawar 25130, Pakistan; dr.muhammadfaizan72@aup.edu.pk

**Keywords:** gut dysbiosis, neuroinflammation, neuronal cells apoptosis, neurotransmitters, natural dietary supplements

## Abstract

Major depressive disorder (MDD) is a mental illness with high mortality, suicide, and relapse rates that could become the leading cause of health problems worldwide by 2030. The microbiota–gut–brain axis involves bidirectional communication between the human gut microbiota and the central nervous system (CNS). The gut microbiome is a complex ecosystem of approximately 100 trillion microorganisms, including viruses, bacteria, and fungi. The gut microbiota has recently been recognized for its impact on various diseases and health concerns. Several factors influence the composition and structure of gut microbes, ultimately affecting human physiology, with the nervous system being particularly vulnerable. The gut–brain–microbiota axis influences several important brain functions through numerous pathways, including vagus nerve signaling, gut microbial synthesis of metabolites, and immune-related chemicals. These factors can influence neurotransmitter activity, neuroinflammation, behavior, and mental health. Despite increased interest, the possibility of modifying the gut microbiota as a therapeutic approach remains unclear. Although numerous studies suggest that microbiota play an important role in many illnesses, the precise mechanisms are yet to be elucidated, and there are currently no evidence-based, microbiota-focused treatments for these illnesses. Recent research indicates that gut dysbiosis (GD) causes increased intestinal permeability (leaky gut), initiates systemic inflammation, and contaminates the blood. Opportunistic microbial metabolites cross the blood–brain barrier, triggering a neuroinflammatory cascade and apoptotic pathways while affecting neurogenesis and neurotransmitters, ultimately resulting in the development of MDD and anxiety. This review examined the factors influencing normal gut microbiota and GD-mediated MDD, as well as possible therapeutic options. The study outlines its objectives and methodological approaches, including the screening and filtering of research on GD-induced depression. Furthermore, it explored the daily use of dietary supplements, revealing new paths for clinical and preclinical research.

## 1. Introduction

According to the American Psychiatric Association, depression is a serious mental health disorder that interferes with daily activities and work routines. This devastating disease affects over 300 million individuals worldwide [1]. Depression, specifically major depressive disorder (MDD), is the most prevalent and second leading cause of disability among adolescents and adults aged 15–45 years. The common physical symptoms of major depression include fidgeting, crying, back pain, headaches, fatigue, tiredness, disturbed appetite, social isolation, insomnia, hypersomnia, unintentional weight change, agitation, and restlessness. Psychological symptoms include difficulty focusing, overthinking, feelings of low self-worth, guilt, hopelessness, frustration, irritation, negative thinking patterns, mood swings, poor concentration, difficulty making decisions, and, in severe cases, thoughts of suicide or death. The development of MDD is influenced by a variety of factors, including metabolic, psychological, environmental, and genetic causes that contribute to the etiology of depression [1,2,3]. Females are more susceptible to MDD than males, with onset typically occurring between the ages of 15 and 30 years [4,5,6,7,8,9,10,11].

According to global estimates, individuals diagnosed with MDD account for more than half of the approximately 800,000 suicides each year. Persons suffering from depression are more than 20 times more likely to commit suicide than the overall population [12,13,14]. The World Health Organization predicts that by 2030, MDD will be a major contributor to the global disease burden. Although MDD affects a large population, its specific causes and development are poorly understood.

For many years, researchers have focused on the monoamine hypothesis, which links depression to differences in brain chemicals, such as serotonin and dopamine [15,16]. Consequently, the most commonly used treatments are standard antidepressant medications that regulate monoamine neurotransmitters. However, these traditional treatments have a low success rate, with only 30–50% of individuals recovering completely, and many experience adverse side effects such as sexual dysfunction and weight gain. Therefore, it is necessary to clarify novel disease mechanisms and uncover new therapeutic targets.

Emerging evidence underlines the critical role of the gut–brain axis (GBA) in neuropsychiatric diseases, including MDD, anxiety, and Alzheimer’s disease [17,18]. The probiotic *Bifidobacterium adolescentis* has been shown to decrease hippocampal proinflammatory cytokine levels (e.g., Interleukin-1β (IL-1β) and tumour necrosis factor-alpha (TNF-α)) and reduce depression-like behaviours in stressed mouse models. This suggests that microbiota modification could be a viable therapeutic option.

The human gut microbiota, which includes approximately 1800 distinct phyla and 40,000 bacterial species, has been linked to several aspects of human health and disease. The microbiota–GBA may affect central functions via the vagus nerve by generating microbial metabolites and immunological mediators that cause changes in neurotransmission, neuroinflammation, and behavior [19,20,21]. Changes in the gut microbiota are associated with various neuropsychiatric conditions, including Parkinson’s disease, autism, schizophrenia, and depression. The precise mechanism by which the gut microbiota causes or modifies neuropsychiatric illness remains unclear [18,22,23,24]. Further research is needed to explain the role of the microbiota–GBA in preventing disease, discovering new therapeutic targets, and enhancing treatments [25].

Gut dysbiosis (GD) occurs when toxic opportunistic microorganisms change the normal composition and structure of the gut microflora. Several clinical and preclinical studies have found significant correlations between GD and severe depression. GD has been reported to alter neuronal transmission (both the hypothalamus–pituitary–adrenal (HPA) axis and afferent fibers of the vagal nerve) associated with local inflammation and increased intestinal permeability, hence facilitating severe depression and anxiety disorders [26,27].

Chronic stress, unknown fear, and sleep disorders activate the immune system via the HPA axis and are common triggers of depression. Increased production of reactive nitrogen species (RNS) and reactive oxygen species (ROS) can damage lipids, nucleic acids, and cellular proteins, as well as disrupt the antioxidant system, resulting in reduced glutathione. ROS also mediates damage to amino acids such as tryptophan and tyrosine. This review examined studies on factors affecting normal gut microflora, altered gut microflora that cause MDD, and prospective therapeutic options for GD-induced depression.

## 2. Materials and Methods

### 2.1. Study Design

This article is a narrative review that examines and critically evaluates the existing evidence on the connection between GD and MDD. The review integrated the results from both preclinical and clinical studies to provide a comprehensive and balanced account of the mechanisms connecting GD with depressive symptoms, as well as the therapeutic potential of microbiota-targeted interventions.

### 2.2. Literature Search Strategy

Major biomedical and multidisciplinary databases (PubMed, Scopus, Web of Science, and Google Scholar) were systematically searched following established procedures [28]. Relevant studies were identified using combinations of keywords, including gut dysbiosis, gut microbiota, major depressive disorder, depression, microbiota–gut–brain axis, neuroinflammation, tryptophan metabolism, oxidative stress, probiotics, prebiotics, and polyphenols. These terms were applied both individually and in combination to capture mechanistic, observational, and therapeutic aspects of the topic.

### 2.3. Eligibility Criteria

Studies were included if they were published in English and were directly relevant to the scope of this review, following established methods [29]. Eligible articles included original research, animal studies, observational human studies, clinical trials, systematic reviews, and meta-analyses. Inclusion required the investigation of mechanistic links between GD and depressive symptoms, the exploration of biological processes involved in the microbiota–gut–brain axis, or the assessment of therapeutic interventions (including dietary strategies, probiotics, prebiotics, and phytochemicals). Studies were excluded if they were not written in English, lacked relevance to GD and MDD, or failed to provide significant evidence toward the review objectives.

### 2.4. Study Screening and Selection

The selection process was conducted systematically. Titles and abstracts were first screened to identify potentially relevant articles, followed by a full-text assessment of eligible studies. Preference was given to studies providing mechanistic understanding, clinically significant results, or strong evidence regarding therapeutic strategies. This approach ensured broad coverage of the available evidence and maintained alignment with the primary focus of the manuscript [30].

### 2.5. Data Synthesis and Thematic Organization

The selected studies were synthesized narratively around key thematic areas in the GD and MDD relationship, including risk factors for dysbiosis, inflammatory and neuroendocrine pathways, microbial metabolites, sleep-related factors, and therapeutic approaches [31]. This structure enabled a coherent interpretation of the evidence and facilitated comparisons across the various types of studies addressing similar biological and clinical questions.

### 2.6. Evidence Appraisal and Interpretation

The literature contained animal models, observational human studies, and a limited number of clinical trials. Following established methods, care was taken to distinguish between levels of evidence [32,33,34]. Emphasis was placed on the power, reliability, and external validity of the results, and associative results were interpreted cautiously to avoid exaggerated causality. This approach ensured scientific balance and accurately reflected the present position of evidence on the role of GD in depression.

### 2.7. Scope and Limitations of the Review Approach

As a narrative review, this study aimed to provide a generalized and integrative synthesis rather than a systematic review or meta-analysis. Although a systematic search and selection process was implemented, the synthesis remained qualitative and subject to variability across the included studies. Consequently, the generalization of findings must be considered in light of the evolving evidence base and the limited number of high-quality clinical trials in this field.

## 3. Factors Affecting Normal Gut Microbiota

**Figure 1:**
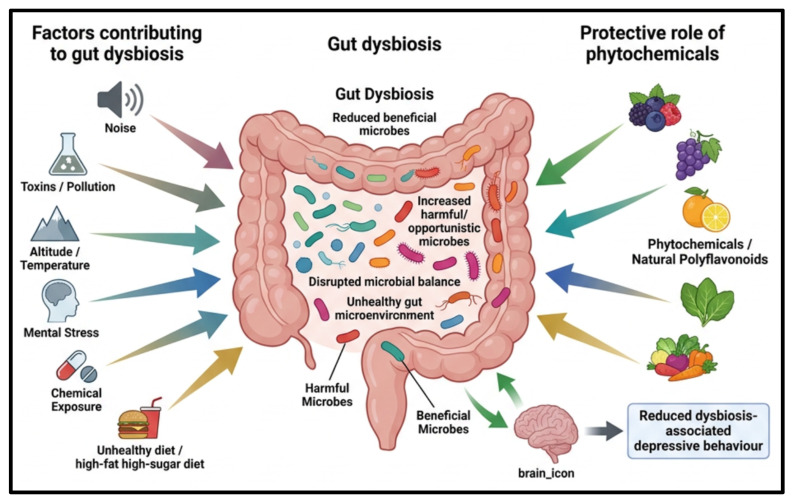
Conceptual summary of gut dysbiosis. Phytochemicals such as natural polyflavonoids may help limit the abundance of harmful microbes and reduce gut dysbiosis-associated depressive behavior.

Various environmental and lifestyle factors can disrupt the normal composition of the gut microbiota, promote GD and increasing susceptibility to neurobehavioral disorders. Factors including noise exposure, environmental toxins and pollution, altitude and temperature stress, mental stress, chemical exposure, and unhealthy diets can alter the balance of beneficial and harmful microbial populations (Figure 1). These disruptions compromise microbial homeostasis and may contribute to dysbiosis-related depressive behavior through downstream effects on gut barrier integrity, immune signalling, and host physiology. Conversely, phytochemicals, particularly natural polyflavonoids, may counteract these alterations by inhibiting detrimental microbes and promoting a healthier microbial equilibrium, offering a promising strategy for preventing or alleviating depressive symptoms associated with GD [35].

### 3.1. Noise

Previous studies on rodents found that acoustic stress increased intestinal permeability, decreased tight-junction protein expression, and induced gastric ulcers and systemic inflammation. Another study demonstrated that aged mice exposed to noise for 30 days had an altered cecal microbiota, characterized by an increase in the Bacteroidetes/Firmicutes ratio [35,36,37,38,39,40,41,42].

### 3.2. Toxins and Environmental Pollution

Air quality and environmental conditions in industrial and war zones are usually unhealthy because industrial chemicals and toxic materials render them unsuitable for human survival. Burn pits or open-air incinerators used by the U.S. military in Iraq and Afghanistan to destroy solid waste produced toxic substances, such as particulates, polychlorinated compounds, and polycyclic aromatic hydrocarbons. Cadmium may affect gut microflora at the phylum and family levels. In a study in which adult mice were exposed to cadmium for 10 weeks, the microbiome was altered and became dominated by opportunistic microorganisms, particularly Bacteroidaceae, which increased serum lipopolysaccharide (LPS) levels. Furthermore, the oral administration of benzo[a]pyrene (BaP or B[a]P) to adult mice for 4 weeks significantly increased the abundance of numerous inflammatory taxa and reduced the abundance of anti-inflammatory taxa (e.g., *Lactobacillus* and *Akkermansia*) [43,44,45,46].

### 3.3. Altitude and Temperature

It is generally recognized that high altitude, particularly above 2500 m, can impact gut microflora composition. Appetite loss, abdominal pain, abdominal gas, vomiting, nausea, and indigestion are adverse effects of hypobaric hypoxia. Intestinal epithelial cells rely on oxygen-saturated blood to perform normal physiological functions and maintain gastrointestinal (GI) motility. A study conducted in the Himalayas revealed an increase in proinflammatory Enterobacteriaceae, along with increased inflammation and decreased *Bifidobacterium* populations. Another study found that soldiers working at high altitudes manifested altered gut microflora. Temperature effects were also examined, demonstrating that acute cold promotes changes in mouse gut microbiota, and positively affects human gut microflora [47,48,49,50,51,52,53,54,55,56,57,58,59].

### 3.4. Mental Stress

Physiological stress can be caused by social isolation and dehydration. This stress creates an imbalance in the body, resulting in inflammation, impaired immunity, altered GI activity, and anxiety-like behavior. The social defeat stress (SDS) model is commonly used in military contexts. Using the SDS model, 2 h of SDS was sufficient to change the mucosa-associated microbes in mice, resulting in a reduction of *Lactobacillus reuteri*. Repeated exposure for 2 h a day over 6 days resulted in increased attenuation of *Lactobacillus*.

The relationship between the gut, brain, and gut microbiota is typically described as bidirectional. Stress-induced activation of the sympathetic nervous system and the HPA axis potentially influences gut microbes and gut-derived signals, impacting the central nervous system (CNS) via the enteric nervous system, spinal and vagal nerves, or blood circulation after crossing barrier systems. Nevertheless, there is debate concerning the relative directionality of this relationship. Some researchers focus on top-down mechanisms, where stress biology modulates intestinal permeability, immune tone, and microbial composition, whereas others emphasize bottom-up mechanisms, where dysbiosis-related metabolites and inflammatory signaling interact with brain activity and behavior. In this regard, the current review considered the gut–brain association to be a dynamic, bidirectional relationship rather than a two-way causal relationship consistently defined across all experimental and clinical settings [47,60,61,62,63,64,65,66,67,68,69].

It was previously recognized that chemical exposure produces severe dysbiosis in the gut, characterized by an increase in the Firmicutes phylum and a reduction in Bacteroidetes abundance. Similarly, the long-term use of corticosterone causes GD. Moreover, recent studies have shown that a lack of intestinal alkaline phosphatase promotes dysbiosis and increases inflammation and intestinal barrier permeability in newborns [70,71,72,73,74].

### 3.5. Diet

The effect of diet on gut microbial composition has been studied during the early stages of colonization; breastfed infants had higher concentrations of *Bifidobacterium* spp., whereas formula-fed infants had higher concentrations of *Bacteroides* spp. Dietary changes may account for 57% of the overall variation in the gut microbiota, whereas genetic factors contribute less than 12%, indicating that diet plays a significant role in shaping it. A Western diet, which is high in sugar and fat, causes dysbiosis and affects the GI tract and immune system. The effect of a high-fat diet (HFD) on mice was studied over three months, revealing a decrease in Bacteroidetes development, alongside significant increases in Firmicutes, Proteobacteria, and Actinobacteria [47,48,49,50,51,52,53,54,55].

### 3.6. Summary of Risk Factors

**Figure 2:**
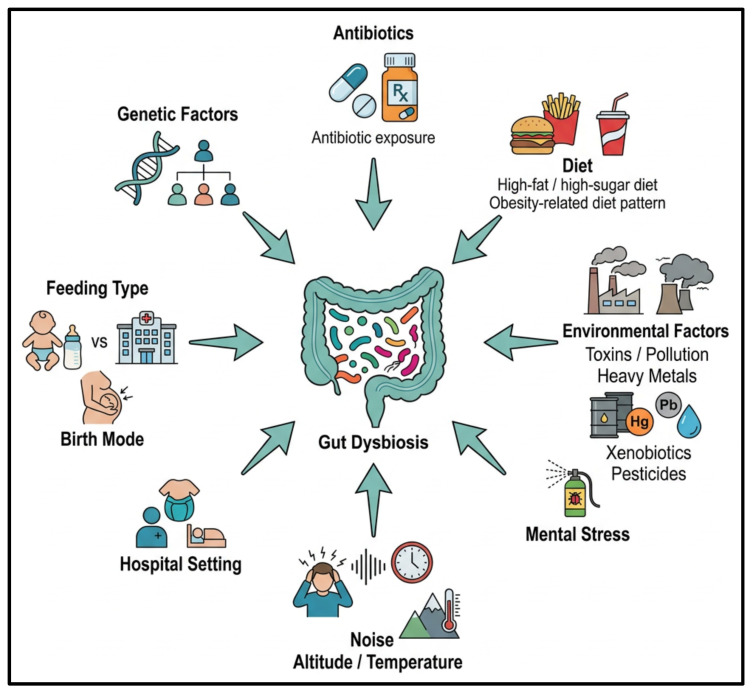
Conceptual summary of common risk factors affecting gut dysbiosis. The figure should be interpreted as a non-hierarchical overview rather than a ranked or pyramidal structure.

Several risk factors affecting the pathophysiology of GD have been proposed (Figure 2). Antibiotic intake has been widely documented to lead to both short- and long-term changes in the gut microbiota. Obese people, as well as those consuming high-fat and high-sugar diets, have shown reproducible gut microbiota modifications. Environmental factors at various life stages influence the development of GD. The type of feeding, hospital setting, and delivery mode are connected to changes in microbiota diversity during infancy. GD is also associated with social pressure and exposure to xenobiotics, heavy metals, and pesticides. Furthermore, research has found that genetics plays a role in the formation of the gut microbiota [56,57,58,59,60].

## 4. Gut Dysbiosis and Its Role in Depression

### 4.1. Gut Dysbiosis Induces Depression Through Its Various Metabolites

*Faecalibacterium prausnitzii* is considered anti-inflammatory and protective, whereas Bacteroides are pro-inflammatory and have been linked to metabolic diseases and chronic pathological conditions. Similarly, the relative abundance of *Prevotella* spp. plays a role in various diseases, including depression-related gut microbial alterations.

**Figure 3:**
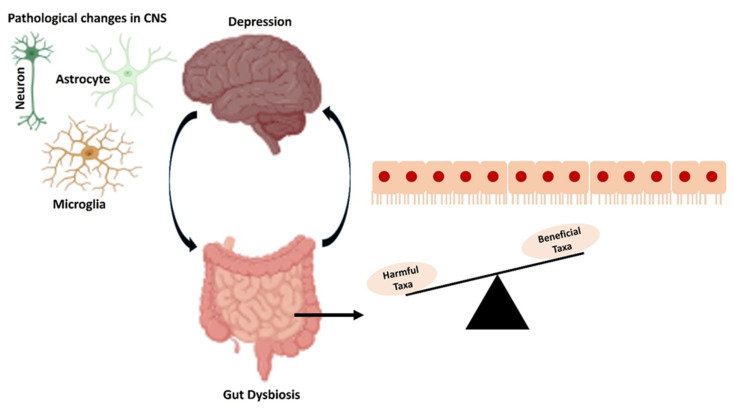
Gut dysbiosis induces depression by releasing various metabolites and activating the microglia and astrocytes in the brain.

The gut microbiota produces several important metabolites. These include short-chain fatty acids (SCFAs), such as isobutyric acid, isovaleric acid, pentanoic acid, acetic acid, propionic acid, butyric acid, and hexanoic acid, which are mainly generated through the fermentation of dietary fibre and prebiotic substrates by intestinal microorganisms [74,75,76]. These metabolites influence the microbiota–gut–brain axis by modulating neurotransmitter synthesis, neuroimmune signalling, intestinal barrier function, and host metabolic activity [75,77,78]. Gut-derived metabolites are also linked with tryptophan metabolism and its downstream pathways, including 5-hydroxytryptamine (5-HT), melatonin [79], kynurenine-related metabolites, and indole derivatives, which influence neuroinflammation, mood regulation, and brain function. Other compounds involved in gut–brain communication include glutamate, γ-aminobutyric acid (GABA), histamine, dopamine, noradrenaline, lipopolysaccharide (LPS), bile acids, catecholamines, and branched-chain amino acids. Furthermore, the gastrointestinal tract secretes several neuroactive and metabolic hormones, including leptin, ghrelin, cholecystokinin, glucagon-like peptide-1 (GLP-1), neuropeptide Y, and peptide YY, which contribute to immune regulation, satiety, glucose homeostasis, anxiety, mood, and behavioural functions [80] (Figure 3).

These metabolites are released either directly or indirectly into the gut by the gut microbiota and subsequently interact with the enteric nervous system and the central nervous system (CNS) through the microbiota–gut–brain axis [79]. Experimental evidence further shows that dietary modulation of the gut microbiota through prebiotics can reduce anxiety- and depression-like behaviours, supporting the role of microbiota-derived metabolites and gut microbial balance in regulating emotional and stress-related responses [79,81]. Typical pathological processes linked to gut dysbiosis-related depressive symptoms include the translocation of pathobionts and toxic metabolites into the bloodstream, depletion of goblet cells leading to decreased mucus secretion and weakening of the mucus layer, destruction of intestinal barrier integrity through the downregulation of tight-junction proteins such as claudin-5 and occludin, and the presence of microbe-associated molecular patterns (MAMPs) [80]. In gut dysbiosis, MAMPs are highly conserved bacterial products, such as LPS and flagellin, which can stimulate inflammatory signaling and contribute to neuroimmune activation [80].

MAMPs stimulate the production of pro-inflammatory cytokines, including IL-18, IL-1β, IL-6, and TNF-α, through inflammatory pathways such as the NOD-like receptor protein 3 (NLRP3) inflammasome and nuclear factor kappa-light-chain-enhancer of activated B cells signaling [80]. LPS can also stimulate toll-like receptor 4 (TLR4), which has been associated with inflammatory responses relevant to major depressive disorder [79]. A leaky mucosal barrier allows LPS and other microbial products to enter the circulation and influence brain function by altering tight junction and adherens junction proteins in brain regions such as the frontal cortex, hippocampus, and striatum, as well as by disrupting blood–brain barrier integrity [80]. In parallel, dysbiosis may disturb tryptophan metabolism by shifting the balance between the kynurenine and indole pathways, thereby influencing immune activation, oxidative stress, neuroactive metabolite production, and CNS-related signaling [82]. Because the kynurenine pathway generates several neuroactive metabolites, its dysregulation has also been linked to neuroinflammation and neurodegenerative processes, suggesting that modulation of the kynurenine pathway may represent an important therapeutic direction for brain-related disorders [83].

**Figure 4:**
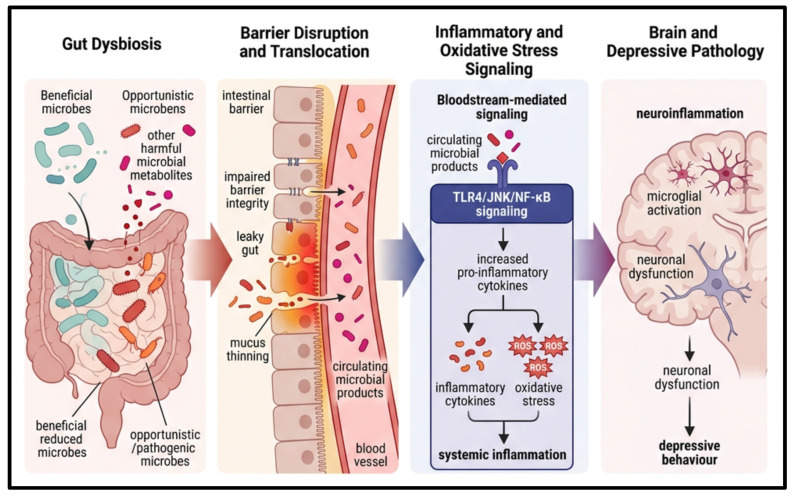
Conceptual summary of opportunistic microbial products that may enter the circulation, impair barrier integrity, and activate inflammatory and oxidative stress pathways such as TLR4/JNK/NF-κB signaling.

Additionally, most human studies report broad changes in microbial diversity or relative abundances, whereas fewer studies identify antagonistic, additive, or compensatory interactions among specific bacterial species in relation to depressive phenotypes [79]. Consequently, discussion of microbial balance should be interpreted with caution because network-level changes are more frequently observed in current research than distinct single-species effects. Future research should combine longitudinal microbiome profiling, metabolomics, and mechanistic validation to determine whether defined microbial consortia have synergistic protective effects or whether specific taxa are biologically resistant to the effects of pro-inflammatory or pathobiont-enriched communities [79,81]. Overall, depressive behaviour appears closely associated with neurochemical signals in gut dysbiosis, altered tryptophan metabolism, intestinal barrier disruption, and a cascade of pro-inflammatory pathways (Figure 4).

### 4.2. Gut Dysbiosis Induces Depression Through Sleep Disorders

**Figure 5:**
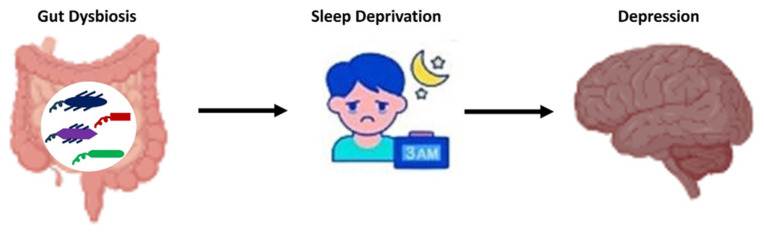
Conceptual summary of how gut dysbiosis may affect sleep quality and quantity and thereby contribute to depressive symptoms.

Recent studies have shown that the microbiota can influence sleep through specific pathways and that sleep disorders play an important role in MDD. The composition of the gut microbiota changes significantly with age, exacerbating major depressive illness caused by sleep disorders. Typical age-related changes in the intestinal microflora include reduced species composition and differences in the quantitative composition of *Bifidobacteria*. During attachment to the intestinal wall, the chemical composition and structure of the colon mucous membrane play a major role. Therefore, bacterial alterations can affect intestinal wall permeability and the release of neurotransmitters, such as GABA and serotonin, which are critical for sleep and brain metabolism. Interestingly, *Bifidobacteria* and *Lactobacillus*, two important beneficial gut microbes, are implicated in GABA production [61,62,63,64,65] (Figure 5). Prebiotics, defined as selectively utilized substrates that confer health benefits, can modulate gut microbiota composition and enhance neuroactive metabolite production, thereby supporting sleep regulation and brain function [84,85,86]. Furthermore, early-life or maternal microbiota modulation by prebiotics may influence long-term metabolic and neurological outcomes [87].

### 4.3. Gut Dysbiosis Induces Depression Through Inflammatory Pathways

Another possible mechanism for neuroinflammation-related depressive symptoms is a change in gut microbes. The pathogenesis of neuroinflammation involves an imbalance in the synthesis and secretion of proinflammatory and anti-inflammatory cytokines, which promotes brain inflammation. Infections, autoimmune diseases, trauma, and other factors can all trigger inflammatory responses.

Recent studies suggest that intestinal flora dysbiosis is a potentially important component of neuroinflammation associated with changes in inflammatory factors. Interventions targeting the gut microbiota—including prebiotics, probiotics, synbiotics, and postbiotics—have demonstrated the ability to modulate inflammatory cytokines and improve related conditions [88,89,90].

Additionally, dietary fibers acting as prebiotics contribute to the production of short-chain fatty acids (SCFAs), which exert anti-inflammatory effects and improve gut barrier integrity [91,92]. Evidence also suggests that specific prebiotics can protect against environmental or toxic stressors by improving physiological resilience [93].

### 4.4. Gut Dysbiosis Induces Depression Through Other Miscellaneous Factors

**Figure 6:**
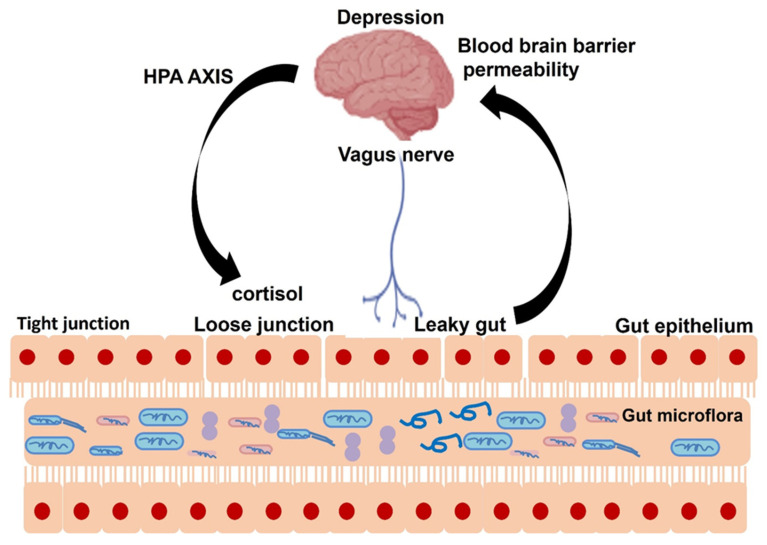
Conceptual description of metabolites from opportunistic microbes disrupting tight junctions, inducing leaky gut, and affecting the central nervous system.

GD changes the composition and quantity of SCFAs, which can mediate depression-like symptoms by regulating tryptophan metabolism and reducing 5-HT levels (Figure 6). These alterations are closely linked to dietary fiber intake and prebiotic availability, which influence microbial metabolic outputs [91,94]. Furthermore, disruptions in microbial metabolism contribute to neuronal dysfunction and depressive behaviors. Prebiotic-mediated modulation of gut microbiota may help regulate microbial metabolites and oxidative stress pathways [84,85]. Reactive nitrogen species (RNS) and ROS may damage cellular proteins, lipids, and DNA. LPS activates oxidative and inflammatory pathways, causing the production of inflammatory cytokines, ROS, and RNS. Malondialdehyde and 4-hydroxynonenal are byproducts of lipid peroxidation associated with oxidative and nitrosative/nitrative stress, both of which have been detected in patients with depression. Oxidative and inflammatory stress also interfere with endogenous antioxidant systems, including melatonin, glutathione, and glutathione peroxidase. The endogenous antioxidant system plays an important role in mitochondrial protection, and its deregulation has been observed in MDD. Nevertheless, opportunistic microbiota may cause depression directly by producing valeric acid (an inverse agonist of the adenosine A1 receptor) and indirectly by increasing kynurenine formation from tryptophan. Furthermore, the translocation of enteral opportunistic microbes into mesenteric lymph nodes activates macrophages and monocytes, resulting in an inflammatory cytokine storm that contributes to depression-like behaviors [95].

### 4.5. Potential Therapeutic Strategies: Special Focus on Phytomedicine

Polyphenols are the most abundant natural compounds found in plants, including fruits, vegetables, tea, coffee, cocoa, and wine. They are classified as flavonoids or non-flavonoids depending on whether they have one or more hydroxyl groups connected to the benzene ring. Flavonoids share a common diphenylpropane carbon skeleton made up of two benzene rings connected by a three-carbon chain. The central chain of three carbon atoms is a component of a closed ring of six carbon atoms known as the pyran ring, which links to the A-benzene ring. Flavonoids are classified into flavones, flavonoids, anthocyanins, flavanones, flavonols, or isoflavones based on the oxidation state of the central pyran ring. Nonflavonoids include phenolic acids, which are subdivided into benzoic acid derivatives (e.g., gallic acid and protocatechuic acid) and cinnamic acid derivatives (e.g., coumaric, caffeic, and ferulic acids).

Dietary polyphenols are phenolic compounds derived from natural sources. In addition to their antioxidant activities, polyphenols possess antidepressant properties. Citrus fruits have a high concentration of the flavonoid hesperidin. Hesperidin can inhibit apoptosis and prevent neuronal degeneration by increasing the levels of phosphoinositide 3-kinases (PI3K), Akt, and rapamycin (mTOR). Apigenin is present in warm tropical vegetables and fruits, particularly celery. By inhibiting p38 and JNK, it penetrates the BBB and exerts an anti-inflammatory effect on BV-2 cells and primary microglia.

Despite the significant potential of polyphenols and other phytochemicals as microbiota-modulating and neuroprotective compounds, available data regarding their antidepressant efficacy in MDD remain largely preliminary. A significant portion of current support is based on in vitro experiments, animal models, or mechanistic studies that demonstrate anti-inflammatory, antioxidant, and gut microbiota-regulating actions. These findings have biological implications but should not be interpreted as conclusive clinical evidence of efficacy in patients with depression. Small Sample sizes, variability in compound type and dose, diverse formulations, differing treatment periods, and a lack of adequately planned randomized clinical trials limit the evidence in humans. Thus, phytochemicals can only be viewed as promising complementary possibilities rather than evidence-based interventions in the treatment of MDD.

## 5. Other Dietary Interventions and Pre/Probiotics

GD and MDD share biological connectivity but exhibit clinical disparities. According to the existing literature, microbiota-specific therapies are not well supported, which should be considered when discussing their translational potential. Dietary interventions, particularly Mediterranean-style diets and probiotic approaches, are widely studied. In comparison, prebiotic interventions are supported primarily by mechanistic and early clinical evidence, including studies combining prebiotics with polyphenol-rich dietary components [84,95,96]. In comparison, the majority of prebiotic and phytochemical interventions are supported primarily by mechanistic data, animal studies, or small preliminary human trials. This distinction is important because biological plausibility alone does not imply clinical efficacy in psychiatric disorders, where symptom heterogeneity, placebo responsiveness, and various confounding factors complicate the interpretation of treatment effects.

Mechanistically, targeting GD in depression is sound. Prebiotics can selectively stimulate beneficial microbes such as *Faecalibacterium prausnitzii*, thereby improving gut homeostasis and metabolic balance [85,97]. Changes in gut microbial flora can affect SCFA synthesis, intestinal permeability, LPS translocation, tryptophan metabolism, serotonergic and glutamatergic signaling, neuroendocrine activity, peripheral and central inflammatory pathways, and oxidative stress. These processes are biologically relevant to the pathophysiology of depression, and they provide a consistent model of how gut microbial imbalances might cause mood dysregulation. However, evidence of therapeutic efficacy should not be mistaken for the presence of such mechanistic links [19,61].

Much of the literature is associative, cross-sectional, or preclinical. Even when animal models demonstrate strong modulation of neuroinflammation, HPA axis responses, hippocampal signatures, or microbial xenobiotic metabolites, the findings are not consistently predictive of antidepressant efficacy of clinical interest in humans. Consistent with this, interpretations in this field should maintain a clear distinction between mechanistic promise and clinical evidence.

Dietary interventions are now among the more clinically plausible options, particularly as better results in depressive symptoms, recovery course, and quality-of-life outcomes have been found in randomized studies following structured Mediterranean-style interventions. Similarly, probiotics are now undergoing rigorous research as microbiota-based interventions and may be seen as a more complex intervention than many other gut-targeted interventions because of their preclinical and rising volume of human trials [20]. However, even substantially stronger sources of evidence are restricted by significant methodological variations. Trials differ significantly regarding diet, baseline nutritional status, strain specificity, dosage, formulation, treatment duration, sample size, co-administered drugs, psychiatric severity, and endpoint selection. Such differences complicate cross-study comparisons and decrease the validity of generalized inferences. As a result, the available evidence on dietary interventions and the use of selected probiotics remains insufficient, inconsistent, and contradictory, failing to provide general clinical guidance for MDD.

At present, prebiotics and phytochemicals must be addressed with caution. Much of the supporting evidence comes from experimental or small-scale human studies, including research on maternal mental health and microbiota interactions [91,96,98]. Their proposed benefits, which include specific enrichment of beneficial taxa, control of microbial metabolite production, alleviation of gut barrier dysfunction, inhibition of inflammasome signaling, and dampening of neuroinflammatory and oxidative pathways, are scientifically relevant and may have therapeutic implications. Nevertheless, most data supporting these interventions remains grounded in experimental systems, mouse stress models, in vitro biotransformation assays, or small-scale human observations, which are currently insufficient to establish an evidence-based psychiatric practice [66,67,68,69,74]. Low bioavailability, uncertainty regarding dose equivalence, dynamic changes in host microbiome composition, and a lack of standardized biomarkers to link microbial shifts to symptomatic improvements all impede clinical translation. Future research must involve large, well-controlled clinical studies integrating longitudinal microbiome profiling, metabolomics, inflammatory biomarkers, neuroendocrine assessments, and conventional psychiatric outcome measures. Future research must involve large, well-controlled clinical studies. Standardized frameworks, including dietary assessment tools and regulatory guidance, highlight the need for consistent evaluation of prebiotic effects [92,94,96].

## 6. Mechanisms by Which Phytochemicals Prevent Gut Dysbiosis–Induced Depression

Licorice plants contain two well-known polyphenols, ferulic acid and hydroxycinnamic acid, which encourage the growth of Bifidobacteria. Previously, an in vitro study showed that bacteria can modify ferulic acid, a fermentation substrate for Bifidobacteria. 5-hydroxytryptophan (5-HTP) is a precursor of 5-HT in humans and has antidepressant properties that are regulated by Bifidobacteria. Several studies have shown that 5-HTP can improve depression by increasing serotonin levels. Other studies have found that Bifidobacteria regulate glutamate, an excitatory neurotransmitter in the CNS, through the glutamatergic synapse pathway. Glutamate activates the *N*-methyl-d-aspartate receptor. It regulates energy reserves, brain plasticity, and synaptic activity, which results in antidepressant effects.

The essential amino acid tryptophan is metabolized via the kynurenine pathway, and several resulting metabolites play vital roles in the pathophysiology of depression [99,100]. Animal studies have shown that modulating polyphenol signaling and its metabolites alters tryptophan metabolism via the kynurenine pathway [101,102]. Resveratrol, a natural polyphenol, was shown to significantly decrease tryptophan levels and increase the kynurenine-to-tryptophan ratio by 1.3-fold after injection in healthy volunteers. Similarly, catechins in black tea increase kynurenine levels, resulting in a higher kynurenine-to-tryptophan ratio in healthy volunteers. Researchers also transplanted the fecal microbiota of patients with depression into germ-free rats, observing changes in tryptophan metabolism, anhedonia, and anxiety behaviors [103,104,105]. The gut flora uses tryptophan to produce serotonin, which is directly related to neuronal signaling. The loss of serotonin, a critical neurotransmitter, is one of the most important blood indicators of severe depression. Serotonin is neuroactive, synthesized peripherally, and plays a key role in neuropsychiatric disorders such as depression [106].

Microglia are important immunological mediators in the CNS, and their activation is linked to clinical psychiatric symptoms and neuroinflammation. Clinical research has shown that many individuals with depression exhibit microglial overactivation, a disorder associated with microglial activation. Studies indicate that the gut microbiota influences microglia, with clear differences observed in the microglial transcriptome between germ-free and specific pathogen-free mice. Genes involved in cell activation were downregulated in the microglia of germ-free mice, indicating that the microbiota plays a role in microglial regulation. Dietary polyphenols have been used to control microglial activation, but the gut microbiota must first produce suitable bioactive polyphenolic compounds. Pomegranate extract contains ellagitannin-like polyphenolic compounds that are poorly absorbed in the small intestine, resulting in low bioavailability. Once they reach the colon, these polyphenolic compounds are metabolized by the gut microbiota into urolithins, a class of bioactive metabolites with a 6H-dibenzo[b,d]pyran-6-one scaffold. Urolithins have neuroprotective effects, reducing microgliosis and amyloid-β plaque deposition. They also improve anxiety-like behavior and memory function. In primary neuronal glia cultures, physiologically active microbially derived epigallocatechin gallate (EGCG) suppresses LPS-induced microglial activation.

The gut microbiota compromises blood–brain barrier (BBB) integrity by inducing peripheral inflammation, which activates the inflammasome, causing depression and further disruption of the gut microbiota. This is known as the microbiota–inflammasome hypothesis of major depressive disorder (MDD). The NLRP3 inflammasome plays an important role in the innate immune system, activating caspase-1 and secreting proinflammatory cytokines IL-1β and IL-18 in response to microbial infections and cell injury [107,108,109,110]. As a result, the NLRP3 inflammasome regulates inflammatory responses, tumorigenesis, and other cellular processes. Animal studies indicate that a high-fat diet (HFD) can activate NLRP3 inflammasomes, leading to low-grade inflammation and an increased risk of colorectal cancer.

*Salmonella typhimurium* and *Escherichia coli* are microbial pathogens known to activate the NLRP3 inflammasome. The activation of this mechanism has yet to be investigated. Gut bacteria can activate this pathway either directly or indirectly. In a previous study, Enterobacteriaceae were found to stimulate IL-1β release through inflammasome signaling following spinal cord injury. These selective Enterobacteriaceae in the microbiome can stimulate monocytes in the injured spinal cord, leading to NLRP3-dependent IL-1β release and intestinal inflammation. Patients with depression exhibited higher levels of NLRP3 gene expression in peripheral blood mononuclear cells, alongside elevated IL-1β and IL-18 in their sera.

**Figure 7:**
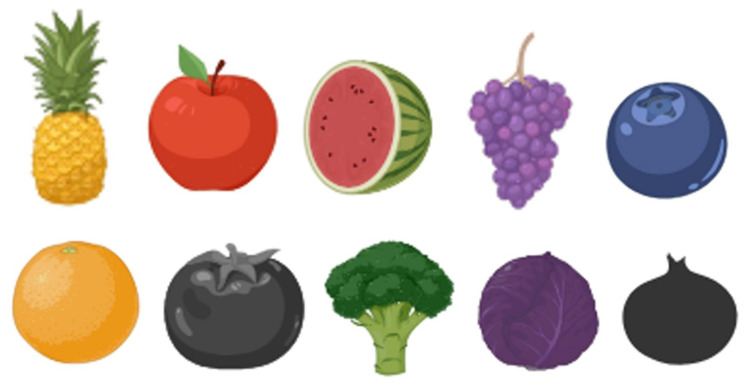
Representative phytocompounds found in vegetables and fruits.

Polyphenol consumption lowers inflammasome activity, thereby reducing depressive symptoms. Lychee seed extracts contain polyphenols such as rutin, quercetin, catechin, and procyanidins [111,112]. Studies show that lychee seed polyphenols stimulate autophagy via the LRP1/AMPK pathway, reducing NLRP3 inflammasome activation [113]. EGCG also affects inflammasome signaling by downregulating NLRP3 gene expression through heme oxygenase-1 regulation. Furthermore, NLRP3 gene knockout reduces depression-like behavior in mice under chronic stress. Various investigations have revealed that EGCG affects inflammasome signaling. EGCG downregulates *NLRP3* gene expression through a mechanism that regulates heme oxygenase-1. Furthermore, *NLRP3* gene knockout reduces depression-like behavior in mice under chronic stress. Previously, researchers investigated the neuroprotective effects of anthocyanins and natural dietary polyflavonoids (Figure 7).

Anthocyanins may protect against LPS-induced neuroinflammation, neurodegeneration, and depressive tendencies. Furthermore, anthocyanins might modulate the TLR4-NF-κB pathway and abrogate LPS-induced neuroinflammation and depression-like behaviors in adult mice. They also inhibit the expression of stress kinases (such as phosphorylated JNK), protect the cellular antioxidant system, and prevent oxidative stress-mediated depression and neuronal cell death. Anthocyanins also inhibit LPS-induced mitochondrial apoptosis through the Cyt/Bax-Bcl2/Casp-3 signaling pathway.

Polyphenols affect neuroendocrine signaling, particularly the HPA axis, which is frequently overactivated during depression. GD may increase stress sensitivity, leading to elevated cortisol levels and disruption of negative feedback regulation. Polyphenols help restore gut microbial balance, normalise HPA axis function, and reduce stress-related behavioural and physiological changes. This normalization enhances emotional resilience and alleviates depressive symptoms. Despite their low bioavailability, polyphenols increase the abundance of immune-modulating bacteria such as *Bifidobacteria* and *Lactobacilli* while suppressing the colonization of pathobionts (Table 1).

## 7. Conclusions and Future Perspectives

Overall, the therapeutic evidence described in this review varies in strength across different interventions. The current evidence for dietary interventions and probiotic techniques is relatively solid, as it relies on human research and, in certain cases, randomized controlled trials. In contrast, several phytochemical and prebiotic strategies primarily depend on mechanistic evidence, animal trials, or a limited number of early-phase human studies. This distinction is vital because promising preclinical results should not be interpreted as conclusive evidence of efficacy for treating patients with MDD. Empirical evidence from the past decade demonstrates that GD is associated with the onset and maintenance of depression, though the strength and significance of this association differ significantly depending on the study design. Changes in the gut microbiota composition can impact microbial metabolite synthesis, neurotransmitter-mediated mechanisms, immunological signaling, and both intestinal and BBB integrity. Concurrently, there is a renewed interest in natural dietary phytochemicals, such as polyflavonoids found in fruits and vegetables, because of their ability to inhibit opportunistic microbial populations, lessen gut barrier dysfunction, and alleviate processes associated with BBB discontinuity.

These compounds can also modulate the effects of serum polysaccharide-mediated systemic and central inflammation, including neuroinflammation and neuronal cell death. Polyflavonoids can promote beneficial taxa, including *Lactobacillus*, *Bifidobacterium*, and *Akkermansia muciniphila*. Gut microbes convert these into bioactive compounds such as phenolic acids and urolithins, which have anti-inflammatory, neuroprotective, and neuromodulatory properties. TLR4/NF-κB ligands, such as LPS, can disrupt tight junction proteins and compromise intestinal barrier integrity. Other metabolites of gut pathogenic microorganisms can increase neurotransmitter release alongside systemic and CNS inflammation. Natural phytochemicals such as polyflavonoids can reduce systemic, central, and neuroinflammatory-related depressive symptoms; modulate neurotrophic factors and neurotransmitters, including dopamine and serotonin; and restore tight-junction proteins and barrier integrity. The low bioavailability of many polyphenols emphasizes the need for optimal dosing and improved delivery methods such as nanoformulations, prebiotic co-administration, and synergistic dietary approaches.

Future research should include large-scale, controlled clinical trials evaluating the gut microbiota, inflammatory markers, and neuroendocrine profiles to determine causality and identify the most effective treatment strategies. The next step will be to determine the additive, antagonistic, or compensatory effects of certain microbial species or consortia, and to integrate microbiome, metabolomic, inflammatory, and neuroendocrine data into a standardized, evidence-based framework. Overall, polyphenols appear to be a biologically viable complementary approach for preventing and alleviating depression associated with GD, but further interdisciplinary research is required before mechanism-oriented clinical translation can be established.

## Figures and Tables

**Table 1:** Phytochemical compounds and their effects related to depression.

Main Compound	Outcome	Source	References
Catechins, Anthocyanins, Proanthocyanins, Flavanols, Epicatechin	Prevent neuroinflammation in the dorsal vagal complex	Cocoa	[114]
Anthocyanins	Significantly increase brain activity with improved working memory and depression-like behavior	Blueberry	[115]
Flavanols, Caffeic acid, Chlorogenic acid	Markedly increases cognitive performance, psychomotor control, and working memory	Coffee	[116]
Fisetin (polyphenol)	Suppresses proinflammatory markers such as TNF-α	Strawberry	[117]
Polyphenol	Increases monoamine and BDNF levels	Peanuts, red grape, wine	[117]
Polyphenol	Elevates serotonin, noradrenaline, and dopamine levels via altering MAO activity	Curcumin	[118]
Polyphenol	Elevates 5-HT and norepinephrine levels in the prefrontal cortex (PFC) and upregulates BDNF levels	Resveratrol	[119]
Epigallocatechin-3-gallate (EGCG)	Confers free radical scavenging and antioxidative properties Green tea treatment can reduce HPA axis hyperactivity in response to stress	Green tea/ epigallocatechin	[120,121]

This table was modified from Sharma Sonali et al., 2022 [122].

## Data Availability

No new data were created for this manuscript. Data sharing is not applicable to this article.
